# Determining current approaches to the evaluation of the quality of healthcare simulation-based education provision: a scoping review.

**DOI:** 10.12688/mep.19758.1

**Published:** 2023-10-05

**Authors:** Rachel Pogson, Helen Henderson, Matt Holland, Agnieszka Sumera, Kacper Sumera, Carl A. Webster

**Affiliations:** 1 School of Medicine, Keele University, Keele, England, ST5 5BG, UK; 2 School of Nursing, Midwifery and Paramedic Practice, Robert Gordon University, Aberdeen, Scotland, AB10 7QE, UK; 3 Library and Knowledge Service for NHS Ambulance Services in England, Bolton, BL1 5DD, UK; 4 Department of Acute Adult Care, University of Chester, Chester, England, CH1 4BJ, UK; 5 European Pre-Hospital Research Network (EPRN), Nottingham, England, UK; 6 East Midlands Ambulance Service NHS Trust, Nottingham Trent University, Nottingham, England, NG11 8NS, UK; 7 Institute of Health and Allied Professions, Nottingham Trent University, Nottingham, England, NG11 8NS, UK

**Keywords:** Simulation, Benchmarking, Healthcare, Evaluation, Quality

## Abstract

**Background:** With an increase in simulation being used in healthcare education, there is a need to ensure the quality of simulation-based education is high. This scoping review was conducted to answer the question: What are the current approaches to the evaluation of the quality of health-care simulation-based education provision?

**Methods:** Databases PubMed, Cochrane, ERIC, CINAHL and Medline were searched in March 2023 to retrieve peer-reviewed healthcare research and review articles written in the English language within the last 20 years. All data were extracted from six studies, themed and presented in the main text and in tabular form.

**Results:** Two scoping reviews, one systematic review and three research articles were included. Three main themes were found: adherence to existing design frameworks, lack of validation of these frameworks and lack of evaluation frameworks, and a proposed evaluation framework. Many of the excluded articles focussed on gaining participant feedback to evaluate simulation activities, rather than evaluating the quality of the design and implementation of the simulation.

**Conclusions:** Benchmarking of current United Kingdom (UK) healthcare simulation against UK and international simulation standards is required to increase its quality, therefore, an agreed UK template framework to evaluate simulation packages is recommended

## Introduction

Simulation has been defined as: “A technique that creates a situation or environment to allow persons to experience a representation of a real event for the purpose of practice, learning, evaluation, testing, or to gain understanding of systems or human actions” ( Lioce *et al.*, 2020).

Simulation-Based Education (SBE) is a student-centred approach underpinned by theories based on constructivism and experiential learning. According to Jeffries (2021), it consists of three sequences: prebriefing, scenario and debriefing. Simulation allows for the varying circumstances involved in patient care and treatment to be better understood and can help in the management of the real situation when it occurs.

Simulation has become an essential approach for educating health professionals ( Gardner *et al.*, 2023). Within the United Kingdom (UK), there has been an unparalleled increase and investment in the use of simulation-based education where it is now being viewed as a key component and cornerstone to healthcare education, particularly in nursing and medical programmes ( Seaton *et al.*, 2019). Several accreditation bodies now require simulation to be embedded within their educational programmes, especially in the United States ( Gardner *et al.*, 2023). Simulation is recognised as an important means of improving patient outcomes through improved learning of evidence-based standards with the ultimate driver being improved quality and safety in healthcare. Simulation is “measurable, focused, reproducible […] and importantly, very memorable” ( DeVita, 2009). 

The benefits of SBE include decreased reliance on training with real patients, allowing for instant feedback for correction of errors, deliberate practice and for directing learning, enhancing the transfer of theoretical knowledge into the clinical context, and ensuring learners are competent before exposure to real patients. As simulation technology continues to develop, scenarios are predicted to improve in the reflection of reality. This includes effective collaborative practice where different professionals learn together by sharing their knowledge, opinions and skills through simulation ( Astbury *et al.*, 2021)

Using simulation-based education requires a standardised, consistent and equitable approach to ensure that quality education is delivered. It requires faculty who can design, facilitate and debrief simulation-based experiences which meet the learning objectives for the scenarios, with the overall aim being to produce students, who when qualified, are resilient, compassionate and safe practitioners and who meet the required standards for their registration ( Watts *et al.*, 2021a)

There is a recognised need to identify alternative ways that can enhance and not replace clinical practice ( Cleaver *et al.*, 2022). With the current issues in healthcare, the growth in student numbers and challenges of capacity in providing quality clinical placements, simulated placements or the use of simulation-based education is being seen as a potential solution to helping to address these problems ( Bogossian *et al.*, 2018). The Nursing and Midwifery Council (NMC) increased the use of simulated practice learning (SPL) in the pre-registration nursing programmes. The maximum number of SPL hours was raised from 300 to 600 this year as a part of the 2300 practice learning hours ( Lewis *et al.*, 2022). The Health and Care Professions Council (HCPC) do not specify a core number of placement hours, nor prescribe whether placement must be clinical or simulated, giving education providers the freedom to apply simulation where they see fit (HCPC, 2021).

If simulation is to be used in these areas, there is a need to ensure that the impact and effectiveness can be consistently demonstrated and that there is a clear evaluation strategy that is benchmarked/linked to standards in simulation. Currently, it seems that there is a dearth of simulation programme evaluation studies ( Kaba *et al.*, 2022). While numerous publications have focused on specific aspects of simulation-based education, such as fidelity or debriefing techniques, few studies have comprehensively examined the evaluation of the entire simulation program. Within the UK there is a recommended SBE standards framework and guidance from the Association of Simulated Practice in Healthcare (ASPiH) ( Purva & Nicklin, 2018). Their framework is currently under review. Internationally there are the revised 2021 International Nursing Association of Clinical Simulation Learning (INACSL) Healthcare Simulation Standards of Best Practice™ ( Watts *et al.*, 2021b) and the updated 2021 criteria for accreditation and certification standards with the Society for Simulation in Healthcare ( SSH), which also focuses on the evaluation and improvement of simulation educational activities. These standards all discuss and highlight the importance of evaluating how simulation is delivered. It is essential to ensure that those who are participating in any simulation experience are being exposed to a properly designed activity ( NMC, 2018; Sahakian *et al.*, 2019) that can safely achieve the required learning outcomes and identify where areas need to be improved.

The lack of evidence around the evaluation of simulation-based education requires further study. As a starting point, this research reviews the recent literature to determine current state of knowledge on this topic.

A preliminary search for previous scoping and systematic reviews on the evaluation of simulation-based education provision was conducted through The Cochrane Database of Systematic Reviews which produced some reviews on simulation, although most focussed on knowledge acquisition for students, rather than evaluation of simulation-based education provision.

## Methods

We used Arksey and O'Malley's (2005) five-stage framework in our scoping review, encompassing research question identification, literature search, study selection, data charting, and result summarisation and reporting. The JBI Manual for Evidence Synthesis (Scoping Reviews) methodology ( Peters *et al.*, 2020) and PRISMA-ScR statement ( Tricco *et al.*, 2018) were utilised. The scoping review protocol was registered on Open Science Framework on 17.3.23 ( https://osf.io/cqshd/).

### Identifying the research question

The PCC (population/concept/context) framework was used to identify the main concepts and inform the research question and search strategy ( Pollock *et al.*, 2023).

***Population*.** Research or review articles that explored the evaluation of the quality of healthcare SBE program provision. The characteristics of participants included healthcare professionals or healthcare students.

***Concept*.** The concept under exploration was approaches to the evaluation of the quality of healthcare SBE provision.

***Context*.** The context was the current approach to the evaluation of the quality of healthcare SBE provision within the healthcare or education setting.

We sought to establish the existing evidence surrounding the standards of simulation-based healthcare education, therefore proposed the following:

a. Objective: *To determine current knowledge about the evaluation of the quality of healthcare simulation-based education provision*.
b. Research question: *What are the current approaches to the evaluation of the quality of healthcare simulation-based education provision?*

### Search for relevant studies

Search terms were devised by the reviewers and peer-reviewed by a research librarian (MH). In March 2023, databases PubMed, Cochrane, ERIC, CINAHL and Medline were searched. PubMed was searched with ‘(Evaluate[Title] OR Quality[Title] OR Standard[Title] OR Review[Title] OR Evaluation[Title]) AND ("simulation based"[Title] OR "simulated practice"[Title])’. Cochrane was searched with ‘"simulation based" OR "simulated practice" in Title Abstract Keyword AND Evaluat* OR Quality OR Standard* OR Review* in Record Title - in Trials (Word variations have been searched)’. ERIC was searched with ‘(Evaluat* OR Quality OR Standard* OR Review*) AND ("simulation based" OR "simulated practice")’. CINAHL and Medline were searched with ‘Evaluat* OR Quality OR Standard* OR Review* “simulation based” OR “simulated practice” with the limiter of ‘peer reviewed’ applied. The terms and Boolean Operators were chosen with the aim of capturing available evidence on the current evaluation of simulation-based education provision. ‘Simulation based’ and ‘simulated practice’ were both used as ‘simulated based practice’ is often used within allied health professions education and simulated practice learning is more often used within nursing education. A bibliographical database was created to store and manage the references.

### Selecting relevant studies

Five members of the research team (RP, HH, AS, KS, and CW) assessed the suitability of the published articles against the following inclusion criteria: articles that explore the evaluation of the quality of healthcare simulation-based education provision. We included any published research and review articles that contained any healthcare field that used simulation-based education, written in the English language. Exclusion criteria were articles that didn’t explore the evaluation of the quality of simulation-based education provision as a whole provision, were not within healthcare/healthcare education fields, were not in the English language, and articles published more than 20 years ago. Excluded articles also included grey literature as it was not considered appropriate to meet the objective and research question for this scoping review.

For standardisation, a sample of the studies were screened by the reviewing team together (RP, HH, AS, KS, and CW). Each source of evidence was then screened by two reviewers independently by title and abstract examination. Any conflict was settled by a third reviewer from the reviewing team. The full-text examination was carried out independently by two of the reviewers. Studies screened at this stage were either included or excluded and conflict resolved by a third reviewer.

### Charting the data

Data from the included articles was aided by a synthesis matrix to organise the information into author(s), year, title, object, study design, and key relevant findings ( Table 1).

**Table 1.** Summary of Articles.

Author(s) and Year	Title	Objective	Study Design	Key Relevant Findings
Cooke *et al.*, 2018	Simulation center best practices: A review of ACS- accredited educational institutes’ best practices, 2011 to present.	To analyse five years of American College of Surgeons accredited educational institutes review reports assessing best practices across standards and criteria in order to identify resources that could be shared among AEIs.	Secondary research; analysis of site reviews.	Good practice significantly impacting quality of SBE provision: A rigorous standardised curriculum development process, and curriculum reviewed by a curriculum review committee, with involvement of educational experts and/or learners. Standardised processes implemented for onboarding new faculty, evaluating faculty, and conducting regular simulation instructor courses. Systematic process for simulator selection/acquisition, and ability to develop or modify custom simulators to meet training needs. Garnering adequate and sustained institutional support, adequate space and equipment for training, efficiency and thoughtful design to meet the unique demands of simulation, and spaces that mimic clinical environments is emphasized Cooke *et al.*, 2018).
Grota & O’Neal, 2020	Using International Nursing Association for Clinical Simulation and Learning Standards to Evaluate the Rigor of High-Fidelity Simulation Learning Experiences.	To share an approach applying INACSL standards to support the substitution of two- hour traditional clinical with one hour of high-quality, rigorous simulation or SLE as determined by faculty governance.	Peer- evaluation of SBE evaluation rubric	In order to achieve high-quality simulation, it is crucial to adopt theoretical and nationally recognized standards. Furthermore, the faculty should have governance over simulation to promote their involvement and ensure quality simulation ( Grota & Neal, 2020).
Salifu *et al.*, 2022	Frameworks for the design, implementation, and evaluation of simulation- based nursing education: A scoping review.	The objectives of this review were to identify frameworks and theories used to guide design, implementation and evaluation of nursing SBE with a focus on the low-resource setting	Scoping review	The lack of a context-specific framework to guide the design, implementation, and evaluation of simulation-based clinical nursing education (SBCNE) in low- resource settings. Of the simulation theory and frameworks included in this review, seven constructs were identified, namely context, background, simulation design, educational practices, facilitator, participant, and outcome ( Salifu *et al.*, 2022).
Smith *et al.*, 2018	The Use of Simulation to Teach Nursing Students and Clinicians Palliative Care and End-of-Life Communication: A Systematic Review.	The objectives were to review the use of SBE to teach communication skills in palliative and end-of-life care.	Systematic review	Debriefing key element of nearly all simulation exercises. Debriefing methods included videotaped session review, question and answer sessions, guided questions, reflective sessions, and the use of published debriefing guides (eg, Debriefing with Good Judgement). Nursing faculty typically facilitated debriefings, but actors, registered nurses, and emergency room residents were also involved. Some authors reported using existing evidence-based frameworks and curricula to guide the development or design of their simulation based learning event (SBLE). All 12 reported conducting pre- and post-evaluations to assess changes in knowledge level, attitudes, and/or self-confidence. Authors reported using a variety of instruments to evaluate the SBLEs, but little to no evidence provided on previous testing or psychometrics. An absence of information that seems critical to assessing the quality and outcomes of the SBLEs in these articles. One is the length of the SBLE. Although the descriptions indicated the SBLEs varied in length, most did not provide sufficient information to assess the actual time involved ( Smith *et al.*, 2018).
Stockert *et al.*, 2022	Simulation-Based Education in Physical Therapist Professional Education: A Scoping Review.	The purposes of this study were to (1) describe and summarize the use of simulation-based education (SBE) with student physical therapists in the international literature and (2) describe the application and integration of standards of best practice (SOBP) for SBE reported in published physical therapy education research.	Scoping review	A summary of the findings relative to the use of 3 INACSL Standards of Best Practice (SOBP)- only 6% of the 182 studies contained all 3 elements: a needs assessment, pre-briefing, and debriefing. Eighty-one of the studies were published prior to dissemination of the INACSL standards in 2016, 17–22 and 101 were published in 2017 or later ( Stockert *et al.*, 2022).
Swart *et al.*, 2019	SASS: South African Simulation Survey - a review of simulation-based education.	The objectives of this study were to describe: i) the state of SBE in anaesthesia within South Africa and a selection of other settings; ii) the learner groups targeted with SBE; iii) the tools used to assess performance during SBE events; iv) the learning objectives targeted; v) the evaluation of the quality and impact of SBE programmes; vi) the resources available within South Africa for SBE; vii) the perceived barriers to the implementation of SBE in South Africa; viii) the attitudes towards SBE; and ix) research and collaboration.	A quantitative online survey	Forty percent of anaesthesia respondents and 38% of non-anaesthesia respondents reported having formal SBE accreditation, but only 11% of all respondents reported attending a formal simulation instructor course. The impact of SBE was evaluated by informal discussions and learner feedback. The methods used for evaluating the impact of SBE: Informal discussion, learner feedback, assessment of learner in simulation, assessment of learner in real situation, patient outcomes ( Swart *et al.*, 2019).

### Collating, summarising, and reporting the results

The extracted articles were collated, summarised, and reported in Table 1. Emerging themes were presented in Table 2.

**Table 2.** Results.

Categories	Evidence
Adherence to existing design frameworks	- In this review, 9 articles reported using an existing framework or curriculum in the design of their SBLEs ( Smith *et al.*, 2018). Some authors reported using existing evidence-based frameworks and curricula to guide the development or design of their SBLE ( Smith *et al.*, 2018). - INACSL SOBP- Only 6% of the 182 studies contained all 3 elements: a needs assessment, pre-briefing, and debriefing. Eighty-one of the studies were published prior to dissemination of the INACSL standards in 2016, and 101 were published in 2017 or later ( Stockert *et al.*, 2022). Pre-briefing was reported in 39.5% of the studies published prior to the INACSL standards compared with 52.5% of studies published afterwards. Debriefing was reported in 54.3% of the studies published before the INACSL standards and in 74.3% of the studies published afterwards. Whereas 8.6% of the studies published prior to the INACSL standards contained all 3 elements, only 4.0% of the studies published after the standards contained all 3 elements ( Stockert *et al.*, 2022). - Common themes among simulation best practices include the following: rigorous application of a standardized curriculum development process, educational expert and/or learner involvement and review by a curriculum review committee ( Cooke *et al.*, 2018).
Lack of validation of design frameworks/lack of evaluation framework	- Authors reported using a variety of instruments to evaluate the SBLEs, but little to no evidence provided on previous testing or psychometrics. The absence of information seems critical to assessing the quality and outcomes of SBLEs in these articles. The limited inclusion of SOBP in SBE research and use of outcome measures that have not been validated limits the production of high-quality research that could be utilized to provide guidance on how to maximize the impact of SBE in physical therapy education ( Smith *et al.*, 2018). - No previous review on simulation in nursing education has identified and described the constructs of frameworks and theories used to guide the design, implementation, and evaluation of simulation in other parts of the world, with a focus on their applicability in a low-resource setting and thus the gaps hindering their application in low-resource settings ( Stockert *et al.*, 2022). - The participant outcome is recorded substantially in the literature and includes increased satisfaction and self- confidence, the acquisition of knowledge, skills, and attitude, as well as behavioural change. What is debated is how SBCNE contributes to knowledge transfer and improvement in patient care and the health system. Studies that linked SBCNE to better patient outcomes have been criticized for their lack of robustness and methodological validity ( Salifu *et al.*, 2022). - Very few of the respondents evaluated the quality or the impact of SBE. This may be due to the relatively new adoption of SBE as a learning tool within South Africa. Participant feedback was mainly used to evaluate SBE quality. This method is potentially subjective and unreliable. Educational impact on more objective indicators such as improved clinical outcomes is difficult to measure or achieve. Currently, informal discussions and learner feedback are most frequently used to evaluate the impact of SBE ( Swart *et al.*, 2019).
Proposed evaluation framework	The faculty adopted Kolb’s theory of experiential learning ( Kolb, 1984) for building the simulation program, the INACSL Standards of Best Practice Simulation (2016) for directing the integration of simulation into curriculum and providing the guidance for simulation development, and the Society of Simulation Healthcare dictionary ( Lioce *et al.*, 2020) for standardizing nomenclature. The rubric, called the SLE evaluation rubric, lists twelve criteria for SLE evaluation on a nominal rating scale. Criterion 12 addresses evaluation. The SLE should have a plan for participant/student evaluation. A limitation of the proposed rubric is that it only evaluates the planning and development of the SLE as 2:1. It does not ensure that SLE is implemented to meet the rigorous requirement of the rubric ( Grota & Neal, 2020).

## Results

**Figure 1.**
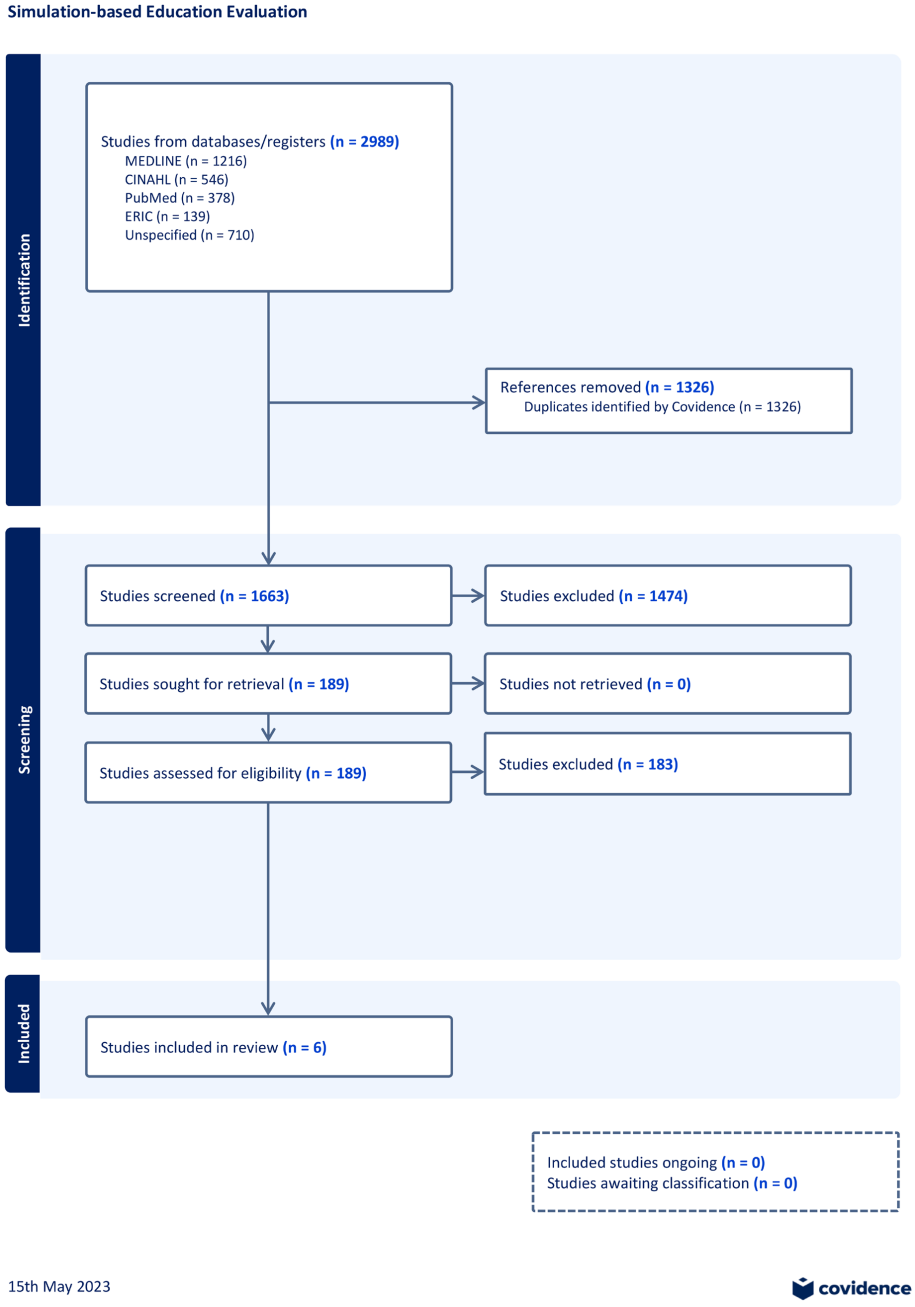
PRISMA flow diagram demonstrating review search results.

The search across databases yielded 2989 publications. After removing duplicates, 1663 papers were left for consideration. Among these, 1474 papers were discarded as they did not meet the inclusion criteria after a review of their titles and abstracts. The remaining 189 papers were assessed for eligibility. Ultimately, six papers were found to meet the inclusion criteria ( Figure 1). Out of the selected studies, two were scoping reviews ( Salifu *et al.*, 2022; Stockert *et al.*, 2022), one was a systematic review ( Smith *et al.*, 2018), one reported evaluation of SBE evaluation rubric and peer-review findings ( Grota & O'Neal, 2020), one reported analysis findings from SBE provision site visits, and one was a quantitative online survey study ( Swart *et al.*, 2019). Of the articles that were screened and excluded, many of these focussed on gaining participant feedback to evaluate simulation activities, rather than evaluating the overall quality of the design and implementation of the simulation. The six included articles were put into three main themes. These were adherence to existing design frameworks ( Cooke *et al.*, 2018; Smith *et al.*, 2018; Stockert *et al.*, 2022), lack of validation of these frameworks and lack of evaluation frameworks ( Salifu *et al.*, 2022; Smith *et al.*, 2018; Stockert *et al.*, 2022; Swart *et al.*, 2019), and a proposed evaluation framework ( Grota & O'Neal, 2020).

### Adherence to existing design frameworks

The systematic review of Smith *et al.* (2018) found some articles that described the use of existing frameworks in the design of simulation-based learning experiences (SBLEs), with a lack of standardisation of what framework was chosen. The scoping review of Stockert *et al.* (2022) found that only six per cent of the 182 studies contained all three elements of the INACSL standards of best practice (SOBP) - a needs assessment, pre-briefing, and debriefing. Interestingly, it was found that 8.6% of the studies published prior to the INACSL standards contained all 3 elements, and only 4.0% of the studies published after the standards contained all 3 elements ( Stockert *et al.*, 2022). In their studied Accredited Educational Institutes, Cooke *et al.* (2018) found a common theme of rigorous application of a standardised curriculum development process, educational expert and/or learner involvement and review by a curriculum review committee. These three studies show differing levels of adherence to existing design frameworks.

### Lack of validation of design frameworks/lack of evaluation frameworks

In their systematic review, Smith *et al.* (2018) found a variety of instruments/measures were used to evaluate simulation-based learning experiences (SBLEs) with little to no evidence of previous testing or psychometrics. They also found little information on the assessment of the quality and outcomes of the SBLEs in the articles they reviewed ( Smith *et al.*, 2018). Stockert *et al.* (2022) found that outcome measures that have not been validated not only reduce the impact of the simulation experience on student learning but also severely limit the production of high-quality research. The scoping review of Salifu *et al.* (2022) found that no previous review on simulation in nursing education had identified and described the constructs of frameworks and theories used to guide the design, implementation, and evaluation of simulation, although their review focussed on low-resource settings. Studies that linked nursing simulation to better patient outcomes had been criticised for their lack of robustness and methodological validity ( Salifu *et al.*, 2022). Swart *et al.* (2019) found in their study that very few of the respondents evaluated the quality or the impact of SBE, which may be due to the relatively new adoption of SBE as a learning tool within South Africa. Simulation participant feedback was mainly used to attempt to evaluate SBE quality and the impact of SBE which could be subjective and unreliable ( Swart *et al.*, 2019). Participant feedback may be useful within simulation evaluation but does not directly evaluate the quality of the activity or adherence to best practice standards. Overall, the literature has shown that there has been limited validation of design frameworks, and although there does appear to be some attempts at evaluating simulation activities, there has been little consistency or standardisation.

### Proposed evaluation framework

The study of Grota & O'Neal (2020) shared an approach applying a new simulation-based learning experience (SLE) evaluation rubric by adopting Kolb’s theory of experiential learning ( 1984) for building the simulation program, the INACSL Standards of Best Practice Simulation ( Sittner *et al.*, 2015) for directing the integration of simulation into curriculum and providing the guidance for simulation development, and the Society of Simulation Healthcare dictionary ( Lioce *et al.*, 2020) for standardising nomenclature. A limitation of the proposed rubric is that it does not ensure that SLE is implemented to meet the rigorous requirement of the rubric. For instance, an SLE can be approved yet, when it is implemented, may be impaired by the lack of available resources such as room assignment, lack of an appropriate manikin, or lack of a trained facilitator ( Grota & O'Neal, 2020). This suggests that an evaluation rubric or framework should also accompany a design framework.

Overall, our scoping review found that, although there is some literature on the adherence to existing design frameworks ( Cooke *et al.*, 2018; Smith *et al.*, 2018; Stockert *et al.*, 2022), comments on a lack of validation in design frameworks and a lack of evaluation frameworks ( Salifu *et al.*, 2022; Smith *et al.*, 2018; Stockert *et al.*, 2022; Swart *et al.*, 2019), and there is one study ( Grota & O'Neal, 2020) that showcases a proposed evaluation tool, there is a paucity of evidence determining current knowledge about the evaluation of the quality of healthcare simulation-based education provision.

## Discussion

### Current status

The aim of this study was to determine the current knowledge of the evaluation of the quality of SBE provisions in healthcare. The results have shown that there is a paucity of research that evaluates the protocols and standards of the delivered SBE and a lack of a standardised framework or template to evaluate SBE. The findings in this review are consistent with the literature. Leighton *et al.* (2020), found no validated tools to evaluate SBE programs and no published literature that comprehensively evaluates SBE programs. Our study shows that this has not changed since 2020. This is significant as stretched healthcare systems continue to face pressure over demand and resources, with increasing healthcare student numbers in clinical placement having a negative impact on the number of clinical hours and the quality of that experience ( Leite *et al.*, 2020). As a result, there has been an increasing focus on replacing or enhancing clinical placement hours with SBE across a wide range of healthcare courses ( Leighton *et al.*, 2020; NMC; Roberts *et al.*, 2019; Wheeler & Dippenaar, 2020). With such a focus on supplementing clinical education with SBE it is important to standardise the approach to evaluating the quality of SBE provisions ( Stockert *et al.*, 2022).

Current trends in evaluating SBE are generally limited to assessing specific learner outcomes, such as a clinical skill or competency, or through the evaluation of the student or facilitator’s perspectives ( Egan *et al.*, 2023; Foronda *et al.*, 2013; Lee *et al.*, 2018). Moreover, this level of evaluation is often self-reported feedback or a self-assessment of the perceived quality. Although this provides important feedback for SBE facilitators, this type of evaluation stops short of benchmarking against agreed standards. This is a crucial step for ensuring the quality of the SBE delivered.

Therefore, the results of the present study indicate that the extent to which education providers adhere to standards for simulation-based education (SBE) is unclear. Moreover, the quality of SBE programs is not currently evaluated or reported in the literature. This finding is concerning as it suggests that the effectiveness and safety of SBE programs are not being fully assessed, which can have potential implications for the quality of healthcare education and patient care outcomes.

### Standards of Best Practice/Frameworks for design and evaluation of quality

Best Practice Standards have been created for the design and development of Simulation-Based Education (SBE), which include the Healthcare Simulation Standards of Best Practice (The INACSL Standards Committee) and the ASPiH standards. The Association for Simulated Practice in Healthcare (ASPiH) developed a set of 21 standards with corresponding guidance in 2016 for centres to use as a framework during simulation design. These standards were created to be broad enough to apply to multiple simulation programs, but more explicit guidance from ASPiH was needed to help centres gauge whether a standard had been achieved ( Egan *et al.*, 2023). In response, Angel *et al.* (2019) developed an audit tool to assess compliance with ASPiH standards. In 2018, ASPiH announced an accreditation process for centres that comply with their standards, claiming that this process will lead to improved simulation quality assurance, organisational networking, formalisation of the simulation process and better meeting of simulation stakeholders' needs ( Angel *et al.*, 2019). ASPiH identifies its audience as "healthcare professionals involved in SBE", and the standards are designed to help them provide quality assurance and improve the delivery of SBE ( Purva & Nicklin, 2018). The ASPiH Standards framework fits with existing practices and priorities of educational bodies and quality assurance bodies, incorporating key elements from quality assurance and standards frameworks published by various bodies ( Purva & Nicklin, 2018).

Without a reliable and validated tool or framework for assessing compliance, it is difficult for individuals, institutions and regulatory bodies to know whether the standards set out by ASPiH and other professional societies are being met. This can ultimately result in the absence of quality assurance, with consequences for patient safety and effective healthcare delivery ( Angel *et al.*, 2019). The Society for Simulation in Healthcare (SSH), which is the largest healthcare simulation accreditation body in the world, has since 2010 offered an accreditation process for centres. This examines the simulation programme’s processes and outcomes in assessment, research, teaching/education and in systems integration and is peer-reviewed. Each area of accreditation has specific standards that are stated, but in addition, in their companion document, there are very detailed descriptions of the standards, which are not meant to be prescriptive but instead are based on the desired outcomes and processes that are the benchmarks of quality healthcare simulation. The aim is that it is a tool that can help identify how best the standards are met. ASPiH also offers an accreditation process for centres that aims to “encompass quality assured educational mechanisms which provide greater credibility [...]” And the organisation sees it as a “quality standard that serves as an authoritative benchmark for assessing performance, rewarding achievement and driving improvement”. Within the UK, there are very few individuals, programmes or institutions that achieved accreditation status from ASPiH ( ASPiH, 2023) or SSH.

Evaluation tools have been suggested, and these have been predominantly structured around the curriculum design standards, such as INACSL ( Grota & O'Neal, 2020; Leighton *et al.*, 2020; Purva & Nicklin, 2018). In the limited literature that does explore the use of a structured approach to SBE program evaluation, there is a lack of adherence to these frameworks, where only parts of the standards have been included, evaluated, or reported on ( Finstad, 2010; Grota & O'Neal, 2020; Stockert *et al.*, 2022). The whole of the SBE provision, rather than parts, need to be systematically assessed, including before simulation, during simulation, after simulation, and the operational efficiency ( Leighton *et al.*, 2020).

The rubric developed by Grota and O'Neal (2020) provides a valuable tool for evaluating the planning and development of high-fidelity simulation learning experiences in nursing. Such rubric could be amended to enable quality assurance of SBE provision ( Grota & O'Neal, 2020).

The absence of a framework or evaluation tool to establish or verify the quality of SBE programs has led to inconsistent reporting of simulation. This could potentially impact research carried out in simulation as there is a consensus that adherence to standard research reporting guidelines would be beneficial ( Cantrell *et al.*, 2017; O'Shea *et al.*, 2020; Weaver *et al.*, 2010).

### Next steps: design frameworks/Evaluation frameworks

ASPiH is due to publish new standards in simulation. We recommend these new standards, along with guidance produced by the Society for Simulation in Healthcare, in terms of their accreditation process for centres that deliver simulation, should be reviewed with key elements identified and then used to generate a suitable framework building on the work by Grota and O’Neal (2020). This could then enable and help to ensure that SBE programmes meet the highest standards of quality and rigour in healthcare education.

## Conclusions

This scoping review has found a paucity of evidence on the evaluation of the quality of delivered healthcare simulation. The literature that does exist looked at adherence to existing design frameworks ( Smith *et al.*, 2018; Stockert *et al.*, 2022; Swart *et al.*, 2019), lack of validation of these frameworks and lack of evaluation frameworks ( Salifu *et al.*, 2022; Smith *et al.*, 2018; Swart *et al.*, 2019), and a proposed evaluation framework ( Grota & O'Neal, 2020).

It is important to address this gap in the literature by developing standards for the evaluation of SBE programs and promoting their implementation in healthcare education settings. This would allow for a more comprehensive understanding of the quality and effectiveness of SBE programs, and enable educators to make informed decisions about the use of SBE in healthcare education.

Now is the time for there to be a greater emphasis in the UK on the standardisation of healthcare simulation. Benchmarking current UK healthcare simulation against UK and international simulation standards is required to increase quality if the simulation is going to be increasingly used to enhance practice and education. An agreed UK template framework to evaluate simulation packages is recommended to measure the quality of this work to be done.

Although this review aimed to determine current knowledge about the evaluation of the quality of healthcare simulation-based education provision, further research, including searching non-healthcare literature, could provide an insight into how other professions benchmark their simulation delivery and evaluation, and these could provide valuable insights for the development of healthcare simulation education provision.

### Limitations

Limitations in this article include the exclusion of grey literature which may limit comprehensiveness. Exclusion was due to concerns about quality and source heterogeneity, prioritising peer-reviewed publications. Secondly, excluding non-English studies may introduce language bias and restrict generalisability. Nonetheless, this study aims to provide valuable insights within its specified scope.

## Data Availability

All data underlying the results are available as part of the article and no additional source data are required.
