# Supplementary material for: Determining current approaches to the evaluation of the quality of healthcare simulation-based education provision: a scoping review
Source: MedEdPublish (2016). 2023 Oct 5;13:207. [Version 1] doi: 10.12688/mep.19758.1 (PMC10767249; doi:10.12688/mep.19758.1)
Supplement: Peer review history

# Reviewer report: Reviewer response for version 1

**DOI:** 10.21956/mep.21172.r35394

**Published:** 2024-01-04

**Authors:** Leizl Joy Nayahangan

**Affiliations:** 1 Copenhagen Academy for Medical Education and Simulation, Center for Human Resources and Education, Copenhagen, Denmark

Thank you for providing the opportunity to review this scoping review, which summarizes current approaches to evaluating the quality of simulation-based education (SBE) provision. I commend the authors for a job well done, and for following a systematic approach to scoping reviews.

 Ensuring the quality of SBE is an area that has not been fully explored and is indeed something that should become a focal point for the simulation community. Aligned with this and the authors findings, we have developed and published an operational rubric (based on implementation science) to evaluate the implementation quality of SBE in healthcare professions education. I do support the need to develop standards and tools to ensure implementation quality simulation-based training programs.

 While I found the paper engaging, I have some constructive comments and suggestions for the authors' consideration in their revision.

  

- In the introduction, the definition of SBE, particularly in the initial paragraphs, appears somewhat restrictive. For instance, the delineation of SBE into three sequences may not universally apply, potentially limiting the scope of the authors' definition. I recommend broadening the definition by incorporating additional references that offer a more comprehensive understanding of SBE.
- Furthermore, a reference is needed for the statement "Simulation is recognized as an important means of improving patient outcomes..." For example: ^1^
- The term "provision" in the context of SBE evaluation is somewhat ambiguous. While it seems the authors intended to cover aspects of development and implementation, the term itself does not explicitly convey this. I propose replacing "provision" with "development and implementation" for clarity and precision.
- Additionally, clarity is needed regarding the authors' definition of "quality" in the context of SBE. What are the benchmarks for quality in healthcare simulation? Have you used these as a framework in your analysis, particularly in the thematic analysis?  Does this align with accreditation as was discussed and suggested as next steps by the authors? 
- The inclusion of only six articles in the scoping review raises questions about the selection process, possibly influenced by the search string. I am curious about the reasons for exclusion, and I recommend incorporating this information into the PRISMA figure for transparency. 
  Thank you for considering these suggestions, and I look forward to seeing the enhanced version of your work.

Are the rationale for, and objectives of, the Systematic Review clearly stated?

Partly

Is the statistical analysis and its interpretation appropriate?

Not applicable

Are sufficient details of the methods and analysis provided to allow replication by others?

Partly

Are the conclusions drawn adequately supported by the results presented in the review?

Yes

Reviewer Expertise:

SImulation-based education; curriculum development; implementation science

I confirm that I have read this submission and believe that I have an appropriate level of expertise to confirm that it is of an acceptable scientific standard, however I have significant reservations, as outlined above.

## References

[1] : Beyond the simulation laboratory: a realist synthesis review of clinical outcomes of simulation-based mastery learning. *Acad Med* .2015;90(11) : 10.1097/ACM.0000000000000938 1553-60 10.1097/ACM.0000000000000938 26375271

# Reviewer report: Reviewer response for version 1

**DOI:** 10.21956/mep.21172.r35390

**Published:** 2023-12-04

**Authors:** Si Qi Yoong

**Affiliations:** 1 Alice Lee Centre of Nursing Studies, Yong Loo Lin School of Medicine, National University of Singapore, 21 Lower Kent Ridge Rd, Singapore

Thank you for this well-written review which has discussed the topic quite adequately and has brought new insights to the evaluation of simulation-based education provision. The methods could be further improved.

 1. Please explain why a scoping review methodology was used and not other review methodologies such as a systematic review.

 2. The section on “collating, summarising, and reporting the results” is too brief. Please refer to the updated guidelines from JBI on what should be described in this section (Pollock *et al.*, 2023 ^1^): “The detail provided by authors should be more than a general statement that they will undertake descriptive statistics, tables, and a narrative summary. Rather, there should be a comprehensive description of the analyses undertaken in order to address each individual review question/objective.” e.g. the content analysis or thematic analysis process should be explained if used.

 3. Table 1 can be improved by including the country where each paper was conducted, the sample size and participant characteristics (and number of studies, type of studies etc. in the case of reviews).

 4. Figure 1: reasons why the 183 articles were excluded were not explained, which should be included in any PRISMA diagram.

 5. The authors only retrieved 6 relevant articles in the review, which is quite little. This could be limited by the search strategy. The search terms for PubMed were only searched based on the title. Usually, the terms should be searched in title/abstract, not just the title. Mesh terms were also not included in the PubMed search strategy. PubMed and Medline are similar databases, I am not sure why the authors searched both. Scopus, a major search database was also not included.

Are the rationale for, and objectives of, the Systematic Review clearly stated?

Partly

Is the statistical analysis and its interpretation appropriate?

Not applicable

Are sufficient details of the methods and analysis provided to allow replication by others?

Partly

Are the conclusions drawn adequately supported by the results presented in the review?

Yes

Reviewer Expertise:

Simulation-based learning in health professions education, systematic reviews

I confirm that I have read this submission and believe that I have an appropriate level of expertise to confirm that it is of an acceptable scientific standard, however I have significant reservations, as outlined above.

## References

[1] : Recommendations for the extraction, analysis, and presentation of results in scoping reviews. *JBI Evid Synth* .2023;21(3) : 10.11124/JBIES-22-00123 520-532 10.11124/JBIES-22-00123 36081365

# Reviewer report: Reviewer response for version 1

**DOI:** 10.21956/mep.21172.r35001

**Published:** 2023-10-25

**Authors:** Amanda Wilford

**Affiliations:** 1 Staffordshire University, Stoke-on-Trent, England, UK

I think the paper identifies a gap in simulation processes.

 One area that would add further strength to the paper is in the introduction - the authors talk about constructivism and experiential learning - linking the two approaches to the Jefferies comment would be useful and the associated references.

 Later on in the proposed framework section Kolb is mentioned, although in a different context adding in a piece that although constructivism was a key element, that it is not there as the authors suggest a common approach.

 The discussion and next steps are really well written with clear indications for practice.

Are the rationale for, and objectives of, the Systematic Review clearly stated?

Yes

Is the statistical analysis and its interpretation appropriate?

Not applicable

Are sufficient details of the methods and analysis provided to allow replication by others?

Yes

Are the conclusions drawn adequately supported by the results presented in the review?

Yes

Reviewer Expertise:

Simulation Healthcare Research particularly design and facilitation

I confirm that I have read this submission and believe that I have an appropriate level of expertise to confirm that it is of an acceptable scientific standard.
